# Oxidative Stress Type Influences the Properties of Antioxidants Containing Polyphenols in RINm5F Beta Cells

**DOI:** 10.1155/2015/859048

**Published:** 2015-10-05

**Authors:** Nathalie Auberval, Stéphanie Dal, William Bietiger, Elodie Seyfritz, Jean Peluso, Christian Muller, Minjie Zhao, Eric Marchioni, Michel Pinget, Nathalie Jeandidier, Elisa Maillard, Valérie Schini-Kerth, Séverine Sigrist

**Affiliations:** ^1^UMR DIATHEC, EA 7294, Centre Européen d'Etude du Diabète, Université de Strasbourg, Fédération de Médecine Translationnelle de Strasbourg, boulevard René Leriche, 67200 Strasbourg, France; ^2^UMR 7200 Laboratoire d'Innovation Thérapeutique, Faculté de Pharmacie, Université de Strasbourg, 67 400 Illkirch, France; ^3^Chimie Analytique des Molécules Bioactives (CAMBA), Institut Pluridisciplinaire Hubert Curien (UMR 7178 CNRS/UDS), 74 route du Rhin, 67400 Illkirch, France; ^4^Departement d'Endocrinologie, Diabète, Maladies Métaboliques, Pôle NUDE, Hôpitaux Universitaires de Strasbourg (HUS), 67 000 Strasbourg, France; ^5^UMR 7175 CNRS, Pharmacologie et Physico-Chimie, Faculté de Pharmacie, Université de Strasbourg, 67 400 Illkirch, France

## Abstract

The *in vitro* methods currently used to screen bioactive compounds focus on the use of a single model of oxidative stress. However, this simplistic view may lead to conflicting results. The aim of this study was to evaluate the antioxidant properties of two natural extracts (a mix of red wine polyphenols (RWPs) and epigallocatechin gallate (EGCG)) with three models of oxidative stress induced with hydrogen peroxide (H_2_O_2_), a mixture of hypoxanthine and xanthine oxidase (HX/XO), or streptozotocin (STZ) in RINm5F beta cells. We employed multiple approaches to validate their potential as therapeutic treatment options, including cell viability, reactive oxygen species production, and antioxidant enzymes expression. All three oxidative stresses induced a decrease in cell viability and an increase in apoptosis, whereas the level of ROS production was variable depending on the type of stress. The highest level of ROS was found for the HX/XO-induced stress, an increase that was reflected by higher expression antioxidant enzymes. Further, both antioxidant compounds presented beneficial effects during oxidative stress, but EGCG appeared to be a more efficient antioxidant. These data indicate that the efficiency of natural antioxidants is dependent on both the nature of the compound and the type of oxidative stress generated.

## 1. Introduction

Oxidative stress can be defined as an imbalance between pro- and antioxidants and is often associated with free radical [1] overproduction and/or defective physiological defence mechanisms resulting in the cell being overwhelmed with oxidizing radicals [2]. This phenomenon involves reactive oxygen species [3], such as superoxide anion (O_2_^−^) [4], hydroxyl radical (OH^∙^) [1], singlet oxygen (^1^O_2_), and hydrogen peroxide (H_2_O_2_) [5]. High concentrations of ROS can cause lipid peroxidation, protein oxidation or denaturation, nuclear acid oxidation, and many other macromolecular changes that can lead to serious cellular damage [6]. Such ROS-related damage has been identified to occur in numerous diseases, including metabolic syndrome, diabetes, multiple types of cancer, Alzheimer's disease, and cardiovascular diseases.

Further, obesity, hyperglycemia, and hyperlipidemia have also been shown to promote oxidative stress through elevated ROS production [7], which is likely due to the higher occurrence of mitochondrial dysfunction and superoxide production that has been associated with fat accumulation [8]. Under normal conditions, enzymatic defence mechanisms [9], such as scavenging by superoxide dismutase [10] and glutathione peroxidase, are active in most types of cells to degrade ROS and prevent cellular damage. However, the antioxidant defence system functioning in insulin producing beta cells, which have been linked to both diabetes and obesity, is known to be very weak [1, 11, 12], making these beta cells highly sensitive to oxidative stress, which can lead to cell death and disease [5]. Notably, the prevention of ROS-related beta cell destruction using antioxidant compounds has been identified to be an effective strategy to delay the onset of diabetes [10, 13, 14].

In fact, several dietary plants that have pharmacological properties shown to prevent apoptosis induced by oxidative stress are under investigation as treatment options for diabetes [9, 15]. Some of these plants appear to utilize antioxidant mechanisms related to their rich flavonoid (polyphenols family) content. The unique chemical structures and redox properties of these polyphenols [16] allow them to scavenge free radicals as well as chelate transition metals and inhibit prooxidant enzymes, such as inducible nitric oxide synthase (iNOS) in macrophages [17]. For example, tea catechins, especially epigallocatechin gallate (EGCG), appear to have antiobesity and antidiabetic properties [11, 18], and the beneficial effects of red wine polyphenols (RWPs) in diabetics have been widely documented [19]. RWPs are qualitatively and quantitatively rich in polyphenols, particularly anthocyanins, flavonol, and stilbene. In general, polyphenols are characterized by antioxidant activity and* in vitro* studies have shown that they act as radical peroxyl scavengers [20]. However, most of these* in vitro* studies were performed using a single model of stress, such as hypoxanthine/xanthine oxidase (HX/XO) [21] or H_2_O_2_ [22, 23], whereby HX/XO was a direct supplier in O_2_^−^, while H_2_O_2_ activated NADPH oxidase or NOS which produce O_2_^−^. In diabetes, in addition to O_2_^−∙^, generated by chronic hyperglycemia [4], other types of ROS are produced during insulin resistance and hyperinsulinism development [24]. Therefore, a single model of oxidative stress does not reflect the full complexity of this disease. In more relevant studies, oxidative stress was induced by multiple mechanisms using cytokines [25], alloxan [26], or streptozotocin [9, 27]. Notably, STZ is an NO donor and induces the formation of several kinds of ROS (e.g., O_2_^−∙^, H_2_O_2_, OH^∙^, and peroxynitrite; Szkudelski, 2001) as well as DNA alkylation and tricarboxylic citric acid (TCA) cycle inhibition, all of which lead to cell damage and death. Thus, STZ can be used to induce multiple levels of oxidative stress in order to more appropriately mimic that which occurs during diabetes* in vivo*.

Obviously the oxidative stress observed during diabetes is complex, and the screening of antioxidant compounds cannot be reduced to the use of a single chemical stress. It is therefore important to validate the antioxidant properties of each antioxidant treatment compound, such as the RWPs, using both simple (single) and complex (multiple) oxidative mechanisms. The aim of this study was to create three* in vitro* models of oxidative stress (one with single radicals produced by HX/XO and two with more complex oxidative reactions using H_2_O_2_ and STZ) in order to assess the antioxidant efficiencies of a RWP extract and a purified extract of EGCG.

## 2. Materials and Methods

### 2.1. Cell Line

Rat insulinoma clone m5F (RINm5F) [28] cells were purchased from the American Type Culture Collection (ATCC, Manassas, USA). Cells were cultivated in Roswell Park Memorial Institute (RPMI-1640) medium supplemented with 10% fetal bovine serum (FBS; Sigma, St. Louis, USA) and 1% antibiotic-antimycotic (ABAM; Gibco, Invitrogen, Grand Island, USA). Cells were grown in a humidified 5% CO_2_ atmosphere at 37°C and trypsinized at 80% confluence using 0.05% trypsin EDTA (Sigma). Medium was refreshed every 48 hours.

### 2.2. Antioxidant Molecules

The RWP extract used in this study was generously given by Dr. M. Moutounet (National Institute of Agronomic Research, Montpellier, France). Red wine phenolic extract dry powder was obtained from French red wine (Corbières AOC) and analyzed by Dr. P.-L. Teissedre (Département d'Oenologie, Bordeaux, France). The extract was prepared as previously described [29]: briefly, phenolic compounds were adsorbed on a preparative column and, then, alcohol desorbed; the alcoholic eluent was gently evaporated; the concentrated residue was lyophilized and finely sprayed to obtain the phenolic extract dry powder. One liter of red wine produced 2.9 g of phenolic extract, which contained 471 mg/g of total phenolic compounds expressed as gallic acid. Phenolic levels in phenolic extract were measured by HPLC. The extract contained 8.6 mg/g catechin, 8.7 mg/g epicatechin, dimers (B1: 6.9 mg/g, B2: 8.0 mg/g, B3: 20.7 mg/g, and B4: 0.7 mg/g), anthocyanins (malvidin-3-glucoside: 11.7 mg/g, peonidin-3-glucoside: 0.66 mg/g, and cyanidin-3-glucoside: 0.06 mg/g), and phenolic acids (gallic acid: 5.0 mg/g, caffeic acid: 2.5 mg/g, and caftaric acid: 12.5 mg/g).

A stock solution of this RWP extract was prepared by diluting 10 mg/mL in a 1 : 1 mixture of distilled water and 100% ethanol. The EGCG extract was a pure form of green tea Teavigo (DSM Nutritional Product, Gland, Switzerland). To confirm its purity, Teavigo was analysed using chromatographic separation on a octadecylsilyl silica gel LC column (l: 0.125 mm; d: 4; 0 mm; Thermo Scientific, France) with spherical particles. Mobile phase consisted of water : formic acid (0.1%, phase A) and methanol : formic acid (0.1%, phase B). A split system was used allowing the HPLC eluate to enter the MS detector at a flow rate of 0.2 mL/min. The injection volume was 20 *μ*L. UV spectral data were acquired at 275 nm (the chromatogram was presented in Supplementary Material available online at http://dx.doi.org/10.1155/2015/859048). The extract was prepared at a 10 mg/mL stock solution concentration which was then diluted in 1× phosphate-buffered saline (PBS), pH 7.4 (Gibco, Invitrogen) as previously described [18]. Cells were cultured for 48 hours before all treatments. The antioxidant compounds (200 to 1000 pg/mM for the EGCG and 100 to 1000 *μ*g/mL for the RWPs) were added to cells seeded in 96-well treated microplates (BD Falcon, Franklin Lakes, USA) at 30,000 cells/well and incubated for 1 hour. The toxicity of the EGCG and RWP extracts was then assessed as described below.

### 2.3. Oxidative Stress Induction and Protective Effects of EGCG and RWPs

Oxidative stress was induced in the RINm5F cells with (1) H_2_O_2_ using a 33% daily prepared solution [30] that was diluted with culture medium to multiple concentrations (1, 10, 25, and 40 *μ*mol/L); (2) a mixture of various HX (mmol/L) to XO (mU/mL) ratios (0.05/2, 0.2/8, 0.25/10, and 0.3/12) isolated from butter milk [22]; and (3) a 40 *μ*mol/L solution of STZ prepared in 0.1 mol/L citrate buffer solution, pH 4.2 (27, 25, and 40 *μ*mol/L, prepared in pH 4.2 citrate buffer solution (0.1 mol/L)) [27]. All products were purchased from Sigma.

To evaluate the toxicity of the EGCG and RWP antioxidants alone, various concentrations (EGCG at 200, 500, and 1000 *μ*g/mL; RWPs at 10, 50, 100, 150, and 200 *μ*g/mL) were added to stressed and unstressed cells and incubated for 1 hour.

### 2.4. MTS Assay

Cell viability was assessed by measuring the mitochondrial activity with the CellTiter 96 AQueous One Solution Cell Proliferation Assay from Promega Corporation (Madison, USA). After treatment, 100 *μ*L of culture medium containing 20 *μ*L of 3-(4,5-dimethylthiazol-2-yl)-5-(3-carboxymethoxyphenyl)-2-(4-sulfophenyl)-2H-tetrazolium (MTS) was added. Cells were incubated for 2 hours at 37°C, in 5% CO_2,_ and the absorbance was measured at 490 nm with a Metertech 960 microplate reader (Metertech Inc., Taipei, Taiwan). The quantity of the formazan product was directly proportional to the mitochondrial activity related to number of living cells. Results are expressed as the percentage of cell viability compared to the appropriate negative controls.

### 2.5. Flow Cytometry

#### 2.5.1. Caspase 8

Oxidative stress often affects the cells very quickly, making it difficult to identify the primary apoptotic effects of the ROS. Therefore, the expression and/or activity of initiator caspases, such as caspase 8 [31], are often used to study apoptosis in this context as they reflect the initial effects of oxidative stress. Here, the activity of caspase 8 was determined by flow cytometry using fluorescent inhibitor of activated caspase 8 (caspase 8 FAM; Millipore, Guava Technologies, Hayward, CA). Briefly, cells were seeded in 96-well treated microplates (BD Falcon, Franklin Lakes, USA) at 30,000 cells/well. After treatment, fresh medium supplemented with 10 *μ*L of caspase inhibitor was added. After mixing, plates were incubated for 1 hour at 37°C and centrifuged for 5 minutes at 300 ×g. Cells were then resuspended and 200 *μ*L of 7-aminoactinomycin D (7′AAD) was added and incubated for 10 minutes at room temperature in the dark before analysis. A Guava Easycyte microcapillary flow cytometer (Millipore) was used with laser excitation at 488 nm in order to detect caspase 8 FAM emission at 517 nm with carboxyfluorescein [6] according to the manufacturer's instructions. For each assay, 200 cellular events were collected. Results were analysed with CytoSoft software (Guava Technologies Inc., Hayward, CA, USA) and are expressed as the percentage of stained cells.

#### 2.5.2. ROS Production

Cells were seeded in 24-well treated microplates (BD Falcon, Franklin Lakes, USA) at 500,000 cells/well. After treatment, 500 *μ*L of fresh medium supplemented with 2′,7′-dichlorofluorescein diacetate (DCFH-DA; Sigma) at final concentration of 5 *μ*M [32] was added. DCFH-DA is deacetylated by a membrane esterase, forming DCFH, which is then transformed by intracellular H_2_O_2_ to a fluorescent molecule [28]. Cells were incubated for 1 hour and then trypsinized. After centrifugation during 10 minutes (500 ×g), pellets were dissolved with 500 *μ*L PBS and 200 *μ*L of the cell solution was transferred to 96-well treated microplates. The suspended cells were then treated with 10 *μ*L of propidium iodide [11] for 15 minutes and assayed for fluorescence by flow cytometry at 530 nm. Assays were analyzed with CytoSoft software and the results are expressed as a percentage of ROS production (DCF-labelled cells) compared to the appropriate negative controls.

### 2.6. Antioxidant Enzyme Expression: Catalase (CAT) and Manganese Superoxide Dismutase (MnSOD)

Protein extracts were prepared from the treated cells using lysis buffer containing 20 mM Tris ultrapure pH 8 (Euromedex, Mundolsheim, France), 137 mM NaCl (Sigma), 1% Igepal CA-630 (Sigma), 31 mM phenyl methyl sulfonide fluoride (Eurobio, Les Ulis, France), and 10% glycerol (Sigma) with protease inhibitor Complete Mini 1X (Roche, Indianapolis, USA). The protein content of each was measured using the methods of Bradford [33].

A 50 *μ*g aliquot of the total protein was then separated on 4–12% Bis-Tris Criterion XT Precast Gels (Bio-Rad), transferred to 0.45 *μ*m nitrocellulose membranes (Bio-Rad), and detected with the following primary antibodies: anti-catalase produced in mouse (Sigma) or anti-MnSOD produced in rabbit (Sigma), each diluted to 1/1000^e^, as well as a 1/5000^e^ dilution of anti-*β*-actin monoclonal antibody produced in mouse. All antibodies were diluted in blocking buffer from the WesternBreeze Chemiluminescent Kit (Invitrogen, Grand Island, USA) overnight at 4°C. Secondary antibody solution (anti-mouse (1/2000) or anti-rabbit (1/4000) coupled to alkaline phosphatase) was incubated with the membranes for 30 minutes with continuous rotation as described in the kit. Membranes were then exposed with a Bio-Rad ChemiDoc XRS System for 600 seconds, and the captured images were analysed using Quantity One software. Expression of CAT and MnSOD proteins was normalized to the quantity of *β*-actin and expressed as a percentage compared to the appropriate negative controls.

### 2.7. Statistical Analysis

Samples were assayed at least three times for each test and the results are given as the mean ± standard error (SEM). Data were analysed with one-way analysis of variance [34] when designated using the Statistica program (Statsoft©, Créteil, France). Treatment differences were subjected to Fischer's test with a 95% significance (*P* < 0.05) threshold.

## 3. Results

### 3.1. Cellular Effects of Various Oxidative Stressors

**Figure 1:**
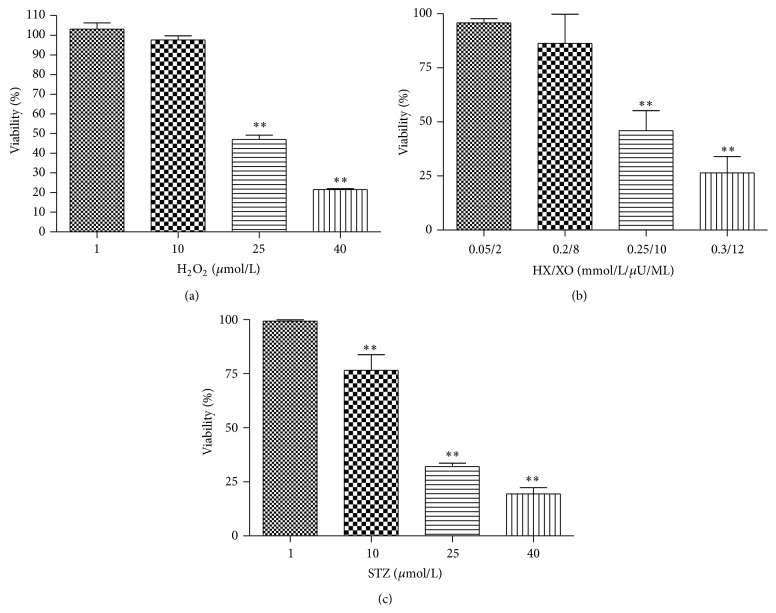
Effects of H_2_O_2_ (a), HX/XO (b), and STZ (c) on RINm5F cell viability. Values are given as the mean ± SEM for three different experiments. *n* = 6; ^*∗*^ *P* < 0.05 and ^*∗∗*^ *P* < 0.01 compared to the control cells.

We first screened the cell viability following treatment with multiple concentrations of each oxidative molecule to determine the concentration of each that induced RINm5F cell death in at least 50% of the culture (Figure 1). H_2_O_2_ appears to induce a significant decrease in cell viability at concentrations above 25 *μ*mol/mL (*P* < 0.01), with the lowest number of viable cells reaching 21.4 ± 0.5% at the 40 *μ*mol/mL concentration (Figure 1(a)). Further, a ratio of 0.25 mmol/L of HX and 10 mU/mL of XO was sufficient to induce a significant loss of cell viability, resulting in only 45.9 ± 9.2% of the cells being viable at this ratio (*P* < 0.01) (Figure 1(b)). Finally, even though the 10 mmol/L concentration of STZ induced a significant decrease in cell viability, leaving only 76.5 ± 0.7% of the cells (*P* < 0.01), a more marked reduction of cell viability similar to that of the other oxidants was observed at 25 mmol/L, which resulted in only 32 ± 1.6% of the cells remaining alive in the culture (*P* < 0.01) (Figure 1(c)). Concentrations that induced a loss greater than 50% (25 *μ*mol/L of H_2_O_2_, a ratio of 0.25 mmol/L of HX and 10 mU/mL of XO, and 25 mmol/L of STZ) were the sole concentrations used in the subsequent analyses to study the antioxidant properties of the EGCG and RWP extracts.

### 3.2. Antioxidant Toxicity Assessment

**Figure 2:**
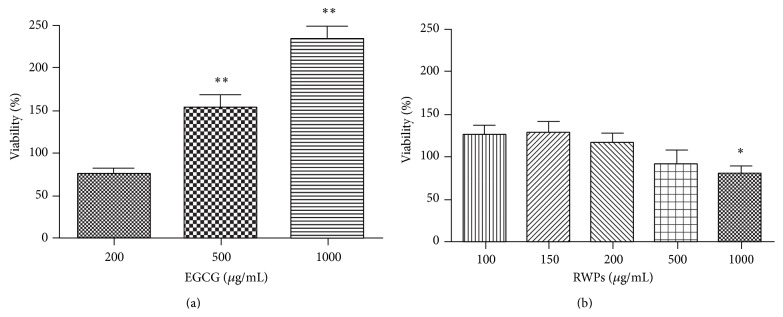
Effects of RWPs (a) and EGCG (b) on RINm5F cell viability. Values are given as the mean ± SEM for three different experiments. *n* = 6; ^*∗*^ *P* < 0.05 and ^*∗∗*^ *P* < 0.01 compared to the control cells.

The potential toxicity of each antioxidant extract on the unstressed RINm5F cells was evaluated by measuring the cell viability in the presence of different concentrations of EGCG and RWPs (Figure 2). RWPs appear to have no effect on cell viability until reaching a concentration of 500 *μ*g/mL, at which point the number of viable cells drops to 92.4 ± 14.7% (not significant). A dose-dependent decreasing trend in cell viability was also observed around this concentration; however, this decrease was not significant until the RWP concentration reached 1000 *μ*g/mL (*P* < 0.05) (Figure 2(a)). In contrast, EGCG was observed to induce a significant increase in cell viability at the 500 *μ*g/mL (152 ± 26%, *P* < 0.01) and 1000 *μ*g/mL (233.5 ± 13.0%, *P* < 0.01) concentrations (Figure 2(b)).

### 3.3. Effects of EGCG and RWPs during H_2_O_2_-Induced Oxidative Stress

**Figure 3:**
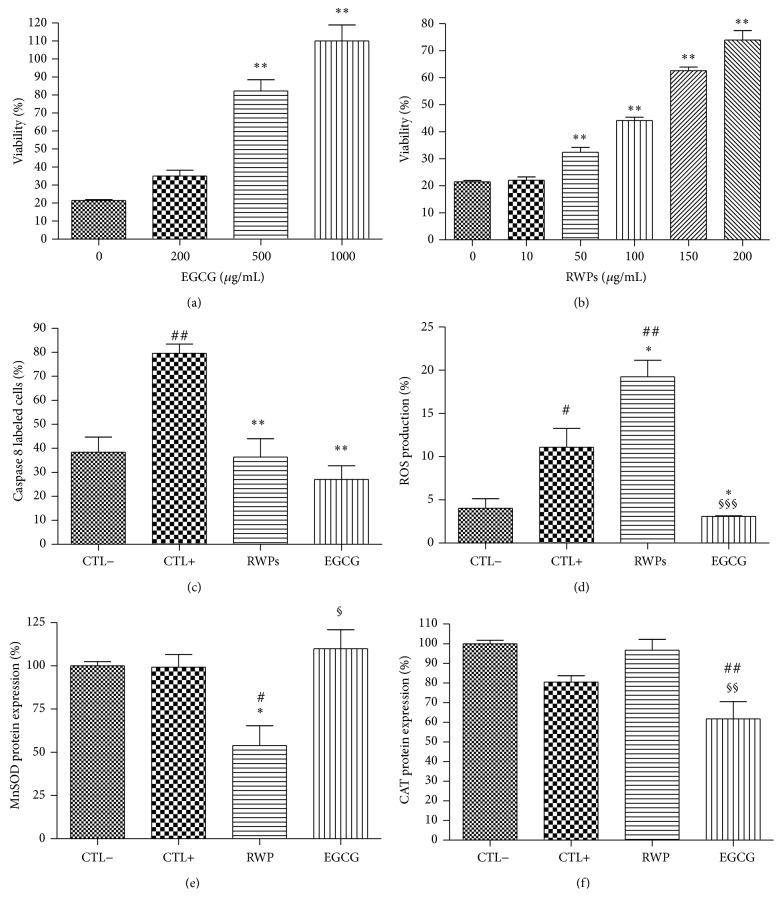
Effects of the antioxidants RWPs and EGCG on cell viability (a, b), caspase 8 activity/apoptosis (c), ROS production (d), MnSOD expression (e), and CAT expression (f) during H_2_O_2_-induced oxidative stress. Values are given as the mean ± SEM for three different experiments. *n* = 6; ^*∗*^ *P* < 0.05 and ^*∗∗*^ *P* < 0.01 compared to the unstressed control cells (CTL−); ^#^ *P* < 0.05, ^##^ *P* < 0.01 compared to the stressed control cells (CTL+); ^§^ *P* < 0.05, ^§§^ *P* < 0.01, and ^§§§^ *P* < 0.001 compared to the stressed cells treated with RWPs.

RWPs were shown to reduce the loss of RINm5F cell viability induced by H_2_O_2_ oxidative stress (Figure 3(a)) in a dose-dependent manner starting at a concentration of 50 *μ*g/mL (32.4 ± 1.8%, *P* < 0.01) and reaching a maximum level at 200 *μ*g/mL (73.9 ± 3.5%, *P* < 0.01), the highest concentration used in this study. Further, 500 *μ*g/mL of EGCG was sufficient to significantly improve cell viability after oxidative stress (82.3 ± 6.2%, *P* < 0.01) (Figure 3(b)). This effect again appeared to be dose-dependent, with the cell viability being completely restored and enhanced at an EGCG concentration of 1000 *μ*g/mL (110 ± 8.9%, *P* < 0.01).

The antioxidant properties of RWPs and EGCG were also confirmed by measuring apoptosis through the evaluation caspase 8 expression using 150 *μ*g/mL of RWPS and 500 *μ*g/mL of EGCG. Both antioxidants appear to reduce the significant increase in caspase 8 activation observed during H_2_O_2_-induced oxidative stress back to levels similar to the unstressed control cells (*P* < 0.01). Surprisingly, the significant increase in ROS production observed during H_2_O_2_ oxidative stress was not reduced by the RWP extract; it actually appeared to induce an increase in ROS production, going from 11.1 ± 2.1% to 19.2 ± 1.9% (*P* < 0.01). On the other hand, EGCG extract significantly reduced ROS production during oxidative stress. In terms of antioxidant enzyme expression during H_2_O_2_-induced oxidative stress, MnSOD (Figure 3(e)) and CAT protein expression (Figure 3(f)) was comparable to that of the unstressed control cells. Further, MnSOD protein expression was significantly reduced by RWPs during oxidative stress (*P* < 0.05), while CAT protein expression was reduced using EGCG (*P* < 0.01).

### 3.4. Effects of EGCG and RWPs on HX/XO-Induced Oxidative Stress

**Figure 4:**
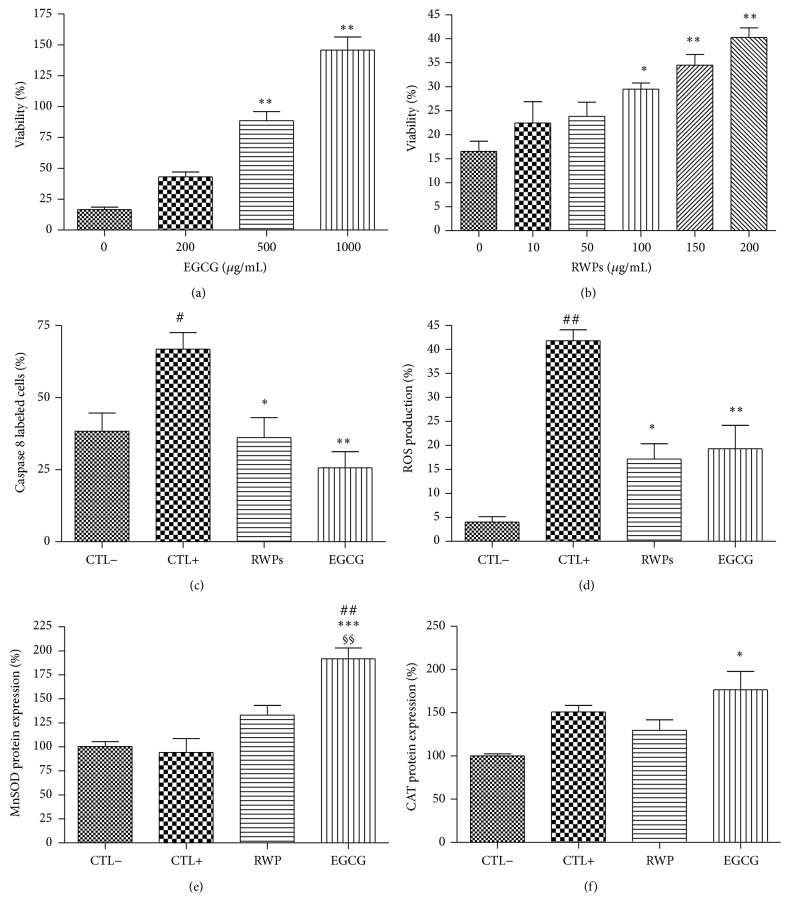
Effects of the antioxidants RWPs and EGCG on cell viability (a, b), caspase 8 activity/apoptosis (c), ROS production (d), MnSOD expression (e), and CAT expression (f) during HX/XO-induced oxidative stress. Values are given as the mean ± SEM for three different experiments. *n* = 6; ^*∗*^ *P* < 0.05, ^*∗∗*^ *P* < 0.01, and ^*∗∗∗*^ *P* < 0.001 compared to the unstressed control cells (CTL−); ^#^ *P* < 0.05, ^##^ *P* < 0.01 compared to the stressed control cells (CTL+); ^§§^ *P* < 0.01 compared to the stressed cells treated with RWPs.

The significant decrease RINm5F cell viability observed during HX/XO-induced oxidative stress was significantly increased when 200 *μ*g/mL of RWP was added, but the percentage of viable cells did not exceed 40.2 ± 1.9% (*P* < 0.01) (Figure 4(a)). The efficiency of EGCG to improve the viability of stress cells was similar to that observed for RWPs at the 200 *μ*g/mL concentration (43.1 ± 3.8%), although this was not significant (Figure 4(b)). However, at higher concentrations EGCG was able to significantly increase cell viability from 16.5 ± 2.1% in the stressed control cells to 88.5 ± 7.4% at 500 *μ*g/mL (*P* < 0.01) and over 100% at the 1000 *μ*g/mL concentration.

Further, the 150 *μ*g/mL and 500 *μ*g/mL concentrations of RWPs and EGCG, respectively, were then used to study their antioxidant properties during HX/XO-induced oxidative stress. It appears that these concentrations of RWPs and EGCG significantly reduce caspase 8 cleavage during HX/XO-induced oxidative stress (*P* < 0.05 and *P* < 0.01, resp.; Figure 4(c)), bringing the caspase 8 activity back down to levels similar to the unstressed control cells. Moreover, both antioxidants significantly reduced the ROS production as well (Figure 4(d)). Notably, only EGCG was observed to increase the protein expression of MnSOD (Figure 4(e)) and CAT (Figure 4(f)) during HX/XO-induced oxidative stress.

### 3.5. Effects of EGCG and RWPs on STZ-Induced Oxidative Stress

**Figure 5:**
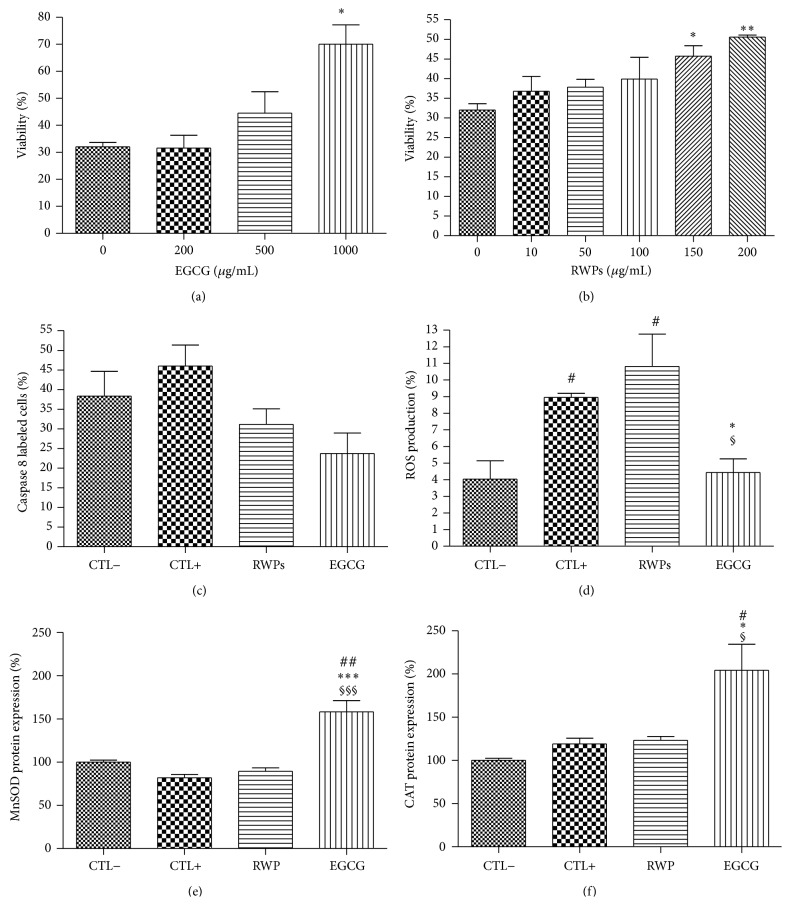
Effects of the antioxidants RWPs and EGCG on cell viability (a, b), caspase 8 activity/apoptosis (c), ROS production (d), MnSOD expression (e), and CAT expression (f) during STZ-induced oxidative stress. Values are given as the mean ± SEM for three different experiments. *n* = 6; ^*∗*^ *P* < 0.05, ^*∗∗*^ *P* < 0.01, and ^*∗∗∗*^ *P* < 0.001 compared to the unstressed control cells (CTL−); ^#^ *P* < 0.05, ^##^ *P* < 0.01 compared to the stressed control cells (CTL+); ^§^ *P* < 0.05, ^§§§^ *P* < 0.001 compared to the stressed cells treated with RWPs.

The significant decrease in cell viability during STZ-induced oxidative stress was slightly opposed by 200 *μ*g/mL of RWPs, which significantly increased the number of viable cells to a mere 50.6 ± 0.5% (*P* < 0.01). Similarly, a 1000 *μ*g/mL concentration of EGCG was needed to significantly increase the cell viability during STZ-induced oxidative stress (60 ± 14%, *P* < 0.05) (Figure 5(b)). For further investigation of the antioxidant properties of the RWPs and EGCG extracts during this type of oxidative stress, we focused on the 200 *μ*g/mL and 1000 *μ*g/mL concentrations of RWPs and EGCG, respectively.

Notably, caspase 8 activation was not observed to increase during STZ-induced oxidative stress; the addition of either of the antioxidants did not significantly change these levels compared to the unstressed or stressed control cells (Figure 5(c)). However, the production of ROS was significantly increased during STZ-induced stress and only EGCG treatment caused a significant reduction of this induced ROS production (*P* < 0.05; Figure 5(d)). The unaltered MnSOD (Figure 5(e)) and CAT (Figure 5(f)) protein expression during STZ-induced oxidative stress was also only affected by the addition of EGCG.

## 4. Discussion

In the present study, we sought to accurately determine the antioxidant properties of two specific natural compounds, RWPs and EGCG. In doing so, we have demonstrated that antioxidant properties depend not only on the nature of the compound itself, but also on the type of oxidative stress induced. These data show, for the first time, that using one model of oxidative stress is not sufficient to make meaningful conclusions concerning the action of an antioxidant, particularly in regard to disease-related oxidative stress.

Complex mechanisms related to glucose autoxidation and hyperinsulinism, which increase ROS production, have been identified in diabetes [5]. Further, the increased level of ROS has been associated with beta cell apoptosis, which, in time, induces insulin dependence [34, 35]. In order to decrease beta cell oxidative stress, nutraceutical approaches have been developed focusing on the screening of plant extract [36–38]. For the initial screening, the efficiency of the bioactive compounds in the plant is currently validated* in vitro* using a single type of oxidative stress [27, 39–41]. Here, in order to provide a comprehensive overview of ROS generated in diabetes, we have utilized three models of oxidative stress to screen our bioactive compounds.

Not surprisingly, the level of cell death induced by each type of oxidative stress studied was similar. However, the level of ROS produced in the cells was found to be higher in the HX/XO model compared to the others. In fact, our data show that there is no correlation between ROS production and the level of cell death, indicating that cell viability alone is not an accurate marker of the oxidative stress response in a cell or tissue. This is in contrast to several previous studies that have linked oxidative stress to cell viability [3, 26, 28]. Notably, the conclusions in these studies were all based on single models of oxidative stress. We chose to measure ROS production in parallel with other markers of the cellular stress response, such as changes in SOD and CAT expression as well as the caspase 8 activity in the cells, in order to evaluate the full effect of the oxidant. According to these results, the cellular defence mechanisms, characterised by SOD and CAT expression, were more activated by the HX/XO-induced stress, which is correlated to the higher level of ROS. Thus, we suggest that antioxidant enzymes likely play a crucial role in cell protection following the introduction of oxidative stress, particularly because the cell viability was comparable to the other conditions as previously described [21, 40]. This phenomenon in the HX/XO experiments could also be explained by the type of radical generated. In fact, the stress induced by H_2_O_2_ and STZ was more complex (ROS generation via NADPH oxidase activation and/or NO synthase) compared to that generated by HX/XO (direct ROS provider), which may result in the latter inducing the cell's response to stress earlier or more efficiently. Additional work is necessary to further elucidate the differing effects caused by the various free radicals.

Using the three stress models outlined here in conjunction with parallel investigations of ROS generation and cellular defence mechanisms to screen bioactive compounds also provided more detailed information on the cellular stress response. For example, in these experiments, EGCG appeared to be a better antioxidant for the three types of different stresses induced, with a decrease in ROS production and an activation of MnSOD and CAT expression, whereas RWPs were shown to be efficient only on the strongest stress (HX/XO) and even then only had a limited influence on antioxidant enzyme expression. Thus, the use of multiple types of free radical stress indicates that EGCG is likely the most efficient scavenger [26] and pharmacological treatment [37] investigated in this study. This is corroborated by a previous study that demonstrated the neuroprotective effects of EGCG involving inhibition of the Fenton reaction and upregulation of several antioxidant enzymes, such as superoxide dismutase and catalase, resulting in the attenuation of oxidative stress [42]. In contrast, the antioxidant properties of RWPs seem to be solely related to scavenging during severe changes in oxidative stress. Moreover, our study suggests that RWPs may be both antioxidant and prooxidant because we observed an increase of ROS production when RPWs were added during H_2_O_2_ oxidative stress. It is well known [43] that polyphenols extract could autoxidize and produce more hydrogen peroxide. In our study, we have demonstrated in this condition that RWPs have no impact on SOD or CAT protein expression but other protective mechanisms could be activated like the stimulation of CAT and SOD activity or a direct action glutathione or thioredoxin. Notably, while we sought to use the same concentration of compound (pure or extract) for both RWPs and EGCG, the composition of these compounds made this impractical. Using HPLC [29], it appears that the raw RWP extract also contains several flavonoids (epicatechin, catechin, and gallic acid) but is found at concentrations that are 100 times less than that of the pure EGCG used in this study. This composition could explain the relatively low efficiency of RWPs shown here. In contrast, the pure EGCG extract was able to activate antioxidant pathways through several mechanisms, which could be due to the high concentration of the flavonoids present.

Regardless of the composition of the compounds, if only one oxidative stress model had been used or only one ROS or cell death assay had been performed, then it is unlikely that the complex nature of these antioxidants would have been uncovered. Therefore, we believe that the sole use of one model or one assay to validate the efficiency of bioactive compounds in the current literature [30, 32] greatly limits the scope and conclusions of these studies. Further, a more precise determination of cell death, ideally using additional apoptosis signalling pathways, like additional caspase activation, antiapoptotic Bcl-xL, and proapoptotic Bax expression [27, 37], as well as further analysis of oxidation-related enzymes could also be utilized to expand our current analysis. Additional work is necessary to determine the full functionality of our multitype oxidative stress model in testing the antioxidant properties of other compounds.

## 5. Conclusion

Here, we have demonstrated that the level of ROS generated by different oxidative stresses was variable and a drug screening using a single kind of stress can introduce bias in the estimation of the antioxidant properties of a compound. Therefore, a combination of several stresses and several cellular and molecular approaches would provide a more accurate experimental text to determine if the compound is an efficient antioxidant. This* in vitro* model also more accurately mimics the* in vivo* biological situation, which will help avoid unnecessary spending of money and time when transitioning a bioactive antioxidant treatment from the context of cell culture to clinical validation. Taken together, this study provides a much needed method for the comprehensive assessment of antioxidants for the treatment of oxidative stress-related diseases.

## Supplementary Material

Chromatographic separation of Teavigo® from DSM demontrated that we have a pure extract of EGCG in this product.

## Abbreviations

7′AAD: 7-Aminoactinomycin D
ABAM: Antibiotic-antimycotic
CAT: Catalase
DCF: Dichlorofluorescein
DCFH-DA: 2′,7′-Dichlorofluorescein-diacetate
DNA: Deoxyribonucleic acid
EDTA: Ethylenediaminetetraacetic acid
EGCG: Epigallocatechin gallate
FAM: Carboxyfluorescein
FBS: Fetal bovine serum
HX/XO: Hypoxanthine/xanthine oxidase
iNOS: Inducible NO synthase
MnSOD: Manganese superoxide dismutase
NO: Nitric oxide
PBS: Phosphate-buffered saline
RINm5F: Rat pancreatic cell line
ROS: Reactive oxygen species
RWPs: Red wine polyphenols
SEM: Standard error of the mean
SOD: Superoxide dismutase
STZ: Streptozotocin
TCA: Tricarboxylic citric acid.
