# Supplementary material for: Oxidative Stress Type Influences the Properties of Antioxidants Containing Polyphenols in RINm5F Beta Cells
Source: Evid Based Complement Alternat Med. 2015 Oct 5;2015:859048. doi: 10.1155/2015/859048 (PMC4609815; doi:10.1155/2015/859048)
Supplement: Supplementary file 1 — Chromatographic separation of Teavigo® from DSM demontrated that we have a pure extract of EGCG in this product. [file 859048.f1.docx]

Teavigo® HPLC

Teavigo®

Injected solution 0.257mg/mL

Chromatographic separation of Teavigo® from DSM (Nutritional Product, Gland, Suisse) was achieved on a octadecylsilyl silica gel LC column (l: 0.125mm; d: 4;0mm; Thermo Scientific, France) with spherical particles. Mobile phase consisted of water:formic acid (0.1%, phase A) and methanol:formic acid (0.1%, phase B). A split system was used allowing the HPLC eluate to enter the MS detector at a flow rate of 0.2 mL/min. at a flow rate of 0.2 mL/min. The injection volume was 20 µL. UV spectral data were acquired at 275 nm.
